# Glucose and HODEs regulate *Aspergillus ochraceus* quorum sensing through the GprC-AcyA pathway

**DOI:** 10.1007/s00018-024-05160-z

**Published:** 2024-05-29

**Authors:** Jing Gao, Huiqing Liu, Yuxin Jin, Yunbo Luo, Kunlun Huang, Zhihong Liang

**Affiliations:** 1 College of Food Science and Nutritional Engineering, China Agricultural University, Beijing, 100083 China; 2 Key Laboratory of Safety Assessment of Genetically Modified Organism (Food Safety), Ministry of Agriculture, Beijing, 100083 China; 3 Beijing Laboratory for Food Quality and Safety, College of Food Science and Nutritional Engineering, China Agricultural University, Beijing, 100083 China

**Keywords:** G protein–coupled receptor, Second messenger, Oxylipin, Mycotoxin, Cell communication

## Abstract

*Aspergillus ochraceus* is the traditional ochratoxin A (OTA)-producing fungus with density-dependent behaviors, which is known as quorum sensing (QS) that is mediated by signaling molecules. Individual cells trend to adapt environmental changes in a “whole” flora through communications, allowing fungus to occupy an important ecological niche. Signals perception, transmission, and feedback are all rely on a signal network that constituted by membrane receptors and intracellular effectors. However, the interference of density information in signal transduction, which regulates most life activities of *Aspergillus*, have yet to be elucidated. Here we show that the G protein-coupled receptor (GPCR) to cAMP pathway is responsible for transmitting density information, and regulates the key point in life cycle of *A. ochraceus*. Firstly, the quorum sensing phenomenon of *A. ochraceus* is confirmed, and identified the density threshold is 10^3^ spores/mL, which represents the low density that produces the most OTA in a series quorum density. Moreover, the GprC that classified as sugar sensor, and intracellular adenylate cyclase (AcyA)-cAMP-PKA pathway that in response to ligands glucose and HODEs are verified. Furthermore, GprC and AcyA regulate the primary metabolism as well as secondary metabolism, and further affects the growth of *A. ochraceus* during the entire life cycle. These studies highlight a crucial G protein signaling pathway for cell communication that is mediated by carbohydrate and oxylipins, and clarified a comprehensive effect of fungal development, which include the direct gene regulation and indirect substrate or energy supply. Our work revealed more signal molecules that mediated density information and connected effects on important adaptive behaviors of *Aspergillus ochraceus*, hoping to achieve comprehensive prevention and control of mycotoxin pollution from interrupting cell communication.

**Supplementary Information:**

The online version contains supplementary material available at 10.1007/s00018-024-05160-z.

## Introduction

*Aspergillus ochraceus* is notorious for its capacity of producing the possibly carcinogenic chemical (group 2B)—ochratoxin A (OTA) [1], and some studies re-identified it as *A. westerdijkiae* [2]. It is an adaptable plant pathogen and significant pollution source that can infect a wide range of foodstuffs during crop production, prolonged storage, and processing. OTA was first extracted from moldy corn, and other susceptible agricultural products, including wheat, rye, sorghum, rice, buckwheat, soybeans and peanuts, are major sources of fungal toxicity [3]. The infection degree of filamentous fungi is influenced by host nutrient composition and functional molecule [4], in addition, stable mycotoxin can accumulate in humans via food chains, attracting worldwide attention [5].

*A. ochraceus* life cycle begins with dispersed dormant spores that grow aggregately after colonizing in suitable habitats, its morphological alteration between spore and hypha, as well as primary metabolism and secondary metabolism, have been shown to depend on population density, known as quorum sensing (QS) [6]. Since discovered the QS phenomenon in *Vibrio fischeri* based on luciferase auto-induction, much more attention has been focused on the bacteria [7]. However as early as 1957, Allen [8] discovered in *Puccinia graminis* that population density could affect fungal morphology, with high density (107 spores/mL) inhibiting spore germination. Analysis of mechanisms, on the one side, it is the adaptive adjustment of fungus species to the overall macro-environment, and on the other, it is the awareness and responsiveness to the micro-environment around cells [9]. The former includes competition for nutrient sources and the latter includes perception of quorum-sensing molecule (QSM). Therefore, accurate perception, effective transmission, and timely feedback of signals are critical for microorganism adaptive survival. In fact, it has been proven that carbon sources, nitrogen sources, typical fungal QSM oxylipins, etc. are all ligands of G-protein-coupled receptor (GPCR) [10].

Transmembrane GPCRs perform the primary function of signals perception by rapidly activating intracellular conjugated guanine nucleotide binding protein (G protein) [11]. Mainly including heterotrimeric G protein (Gαβγ) and small GTPase (RasA), which interact with respective effectors through conformational changes to activate or inhibit specific downstream pathways [12] In 2000, Palczewski et al. [13] solved the 3D fine crystal structure of bovine rhodopsin binding with 11-cis-retinaldehyde complex which was the first GPCR to be deconstructed, providing a revolutionary guiding value for comprehending signal transduction and drug creation based on its structure. GPCRs are classified according to their structures and ligands [14]. Lafon et al. [15] explored *Aspergillus* genomes in silico and divided 15 GPCRs into nine classes. Affeldt et al. [16] conducted the first GPCRs genome-wide assessment in *A. flavus*, confirming their function by constructing mutant strains of each *gpr* gene, involving in morphogenesis, mating, nutrient sensing, secondary metabolism, stress agents, and lipid responses. They also found existing crosstalk in downstream effect pathways of GPCRs [17], including adenylate cyclase-cyclic adenosine monophosphate-protein kinase A (AcyA-cAMP-PKA) [18], inositol triphosphate (IP_3_)-Ca^2+^-diacyl-glycerol-dependent protein kinase C (PKC) [19], and mitogen-activated protein kinase (MAPK) [20]. Previous researches have pointed in the right direction and laid a solid foundation.

Here in *A. ochraceus*, we delve into the quorum sensing mechanism. According to the density-dependent behaviors confirmed the density threshold, and through transcriptome alignment of low and high densities, we discovered that the density information was perceived by GprC and transmitted by the second messenger cAMP. The GprC-AcyA pathway is responsible for intraspecific and interspecific communication mediated by QSM hydroxyoctadecadienoic acids (HODEs), as well as glucose activation on development and metabolism. However, the regulation of secondary metabolism and growth demonstrated by GprC-AcyA is multifaceted. Transcriptome alignment of wild-type (WT), AcyA deficient (Δ*acyA*), and GprC deficient (Δ*gprC*) strains were performed. On the one hand, we found these two elements can directly act on regulatory factors to affect genes of OTA biosynthesis and sporulation, while on the other hand, the GprC-AcyA pathway first affects primary carbon metabolism, which supplies energy and substrates, then influences growth, reproduction and secondary metabolism of fungus.

In conclusion, this work demonstrates the comprehensive regulatory function of GprC-AcyA pathway in fungal QS. Research into the signal perception and response mechanism of *A. ochraceus* will not only fill present theoretical gaps, but also extend to a broader spectrum of species. This can accomplish artificial control of fungal activity, providing targets for fungus pollution control and opening up new opportunities.

## Results

### *Aspergillus ochraceus* performs typical quorum sensing phenomena

**Fig. 1:**
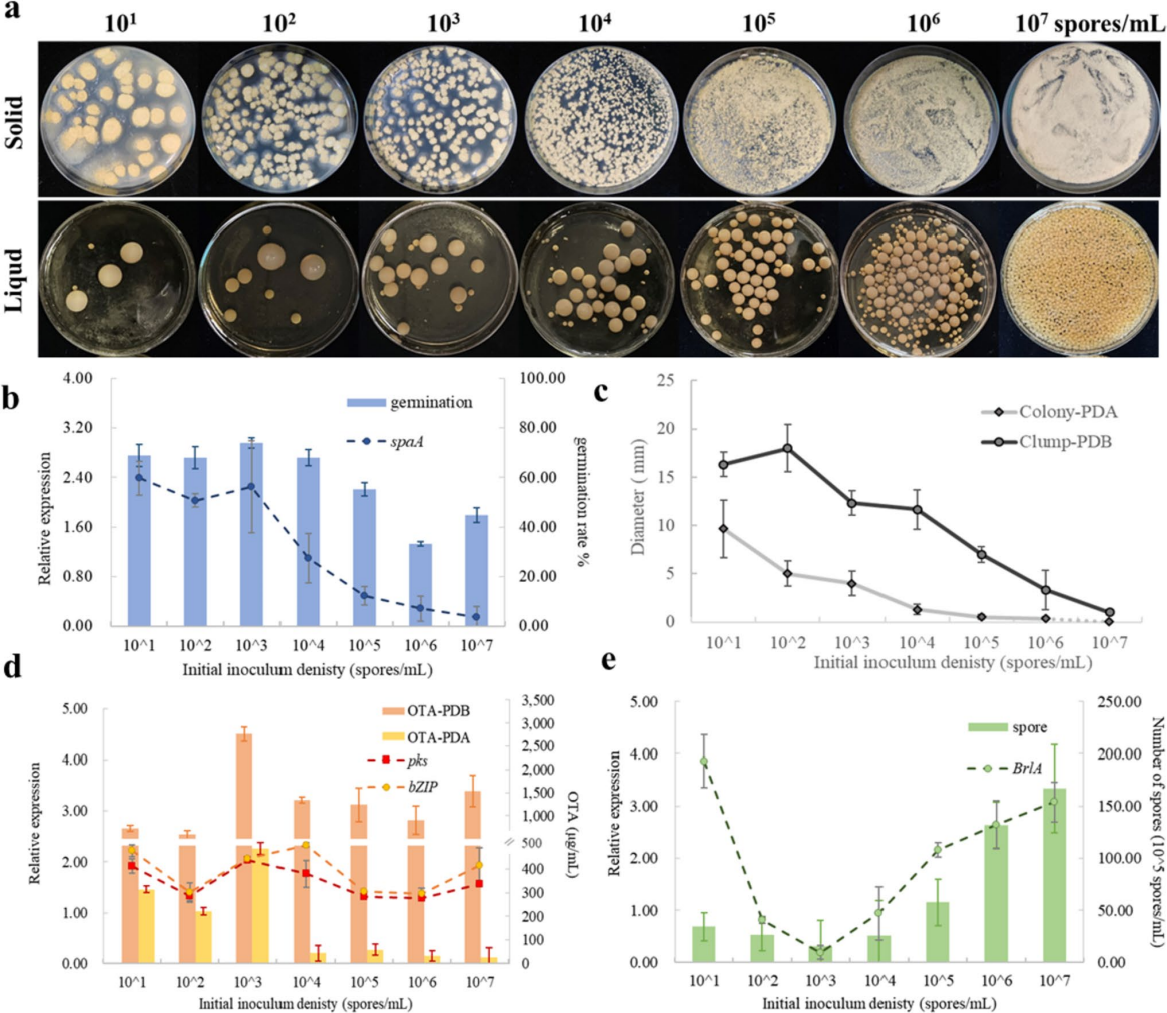
Density-dependent activities of *A. ochraceus*. **a** Growth in solid PDA (upper) and liquid PDB (bottom) medium. **b** Germination rate and the expression of polocyte forming gene *spaA* after culturing 14 h in PDB. **c** Diameter of colony and clump pellet, and **d** OTA production as well as the expression of *pks* and *bZIP* located in the OTA biosynthetic gene cluster after culturing 72 h in PDA and PDB. **e** The quantity of spores and the expression of sporulation regulatory factor gene *brlA* after culturing 120 h in PDA. Dashed lines represent genes, and columns represent physical substances

There is a certain link between *Aspergillus* spp. behavior and population density, here in *A. ochraceus*, we first determined the spores germination and sampling time of *A. ochraceus* according to the growth curve (Figure S1 and S2), and then evaluated the QS phenomenon among the 10^1^ ~ 10^7^ spores/mL initial density range in both solid and liquid culture substrates (Fig. 1a). Observing from spores, found that the germination rate remained stable in a lower density range but decreased with the increase of population density, and the *spaA* gene that encoding polocyte forming began to decline after 10^3^ spores/mL (Fig. 1b). The size of aggregates was always negatively correlated with population density (Fig. 1c), which was presumed to be limited by space. Secondary metabolite OTA with the maximum yield at 10^3^ spores/mL and the expression of OTA biosynthesis initiation gene *pks* followed a consistent trend with OTA production, while with the density increasing the expression of gene *bZIP* fluctuated slightly, which was responsible for specific regulation of most of the genes in this cluster (Fig. 1d). However, for asexual reproduction, the expression of sporulation gene *brlA* was lowest and produced the fewest spores at 10^3^ spores/mL (Fig. 1e). These phenomena indicating that the density information has an opposite effect on fungal development, and 10^3^ spores/mL is the density threshold for *A. ochraceus* QS behavior shift.

### GprC-AcyA pathway responsible for sensing and transmitting density information

**Fig. 2:**
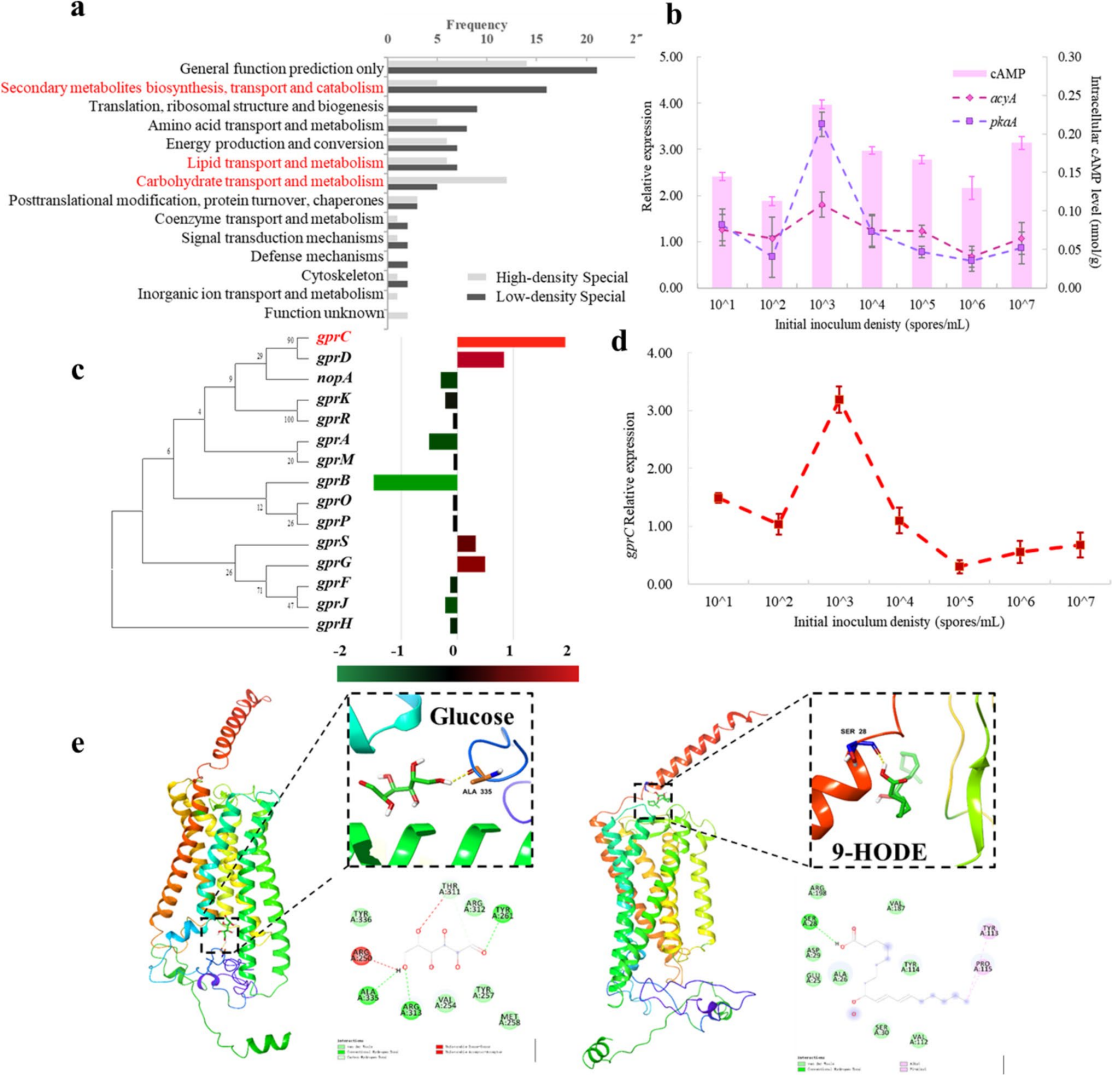
Mechanism analysis of *A. ochraceus* responding density information. **a** Statistics of specific expression genes between representative low-density (experimental group, 10^3^ spores/mL) versus high-density (control group, 10^6^ spores/mL). **b** The density dependence of cAMP-cAMP-PKA pathway, including the intracellular cAMP level as well as expression of *acyA* and *pkaA*. **c** Gene expression difference of GPCR family. **d** The density dependent expression of *gprC*. **e** Molecular docking simulation of the receptor GprC with the ligand glucose and 9-HODE, with the binding amino acids (upper) and chemical structure (bottom) zoomed on the right

Fungal quorum sensing necessarily involves complex intracellular regulation, we compared the transcriptomes of low-density (10^3^ spores/mL, experimental group) and high-density (10^6^ spores/mL, control group) to analyze the mechanism of density dependent shifts in *A. ochraceus*. There was more secondary metabolism related genes specifically expressed under low-density conditions, and various carbohydrate metabolisms were more active under high-density (Fig. 2a). In both density, there were specific expression genes that involved in lipid transport and metabolism, as well as membrane transport function (Fig. 2b), which may be related to the response to signal carrying density information.

Only the AcyA-PKA pathway was significantly up-regulated among intracellular major cascade signaling pathways (Supplementary data 1), we found that gene expression of adenylate cyclase (*acyA*) and protein kinase A (*pkaA*), as well as cAMP level were all density dependent and peaked at the 10^3^ spores/mL like OTA (Fig. 2b), implying that the “AcyA-cAMP-PKA” pathway regulates OTA biosynthesis within the cell, which is located downstream of the GPCR family. Screening membrane receptor that perceive signals, only GprC was significantly up-regulated at low-density (Fig. 2c), and *gprC* expression was also density dependent that peaking at 10^3^ spores/mL while decreasing greatly as density increased (Fig. 2d). According to phylogeny that GprC was classified as class III (Figure S3), which is the carbon sensor and possible oxylipin receptor [21]. We simulated the molecular docking of GprC with glucose and 9-HODE, the binding energy of these two ligands were both -4.6 kcal/mol and mainly via hydrogen bond interactions, but the binding site with GprC protein were different, glucose with the ALA335 and 9-HODE with the SER28 (Fig. 2e), which suggest that GprC may bind various ligands at certain stages specifically, resulting in different effects on cell. However, these theoretical conjecture still lack proof.

### Transcriptomic showed that GprC and AcyA regulate development at gene level

**Fig. 3:**
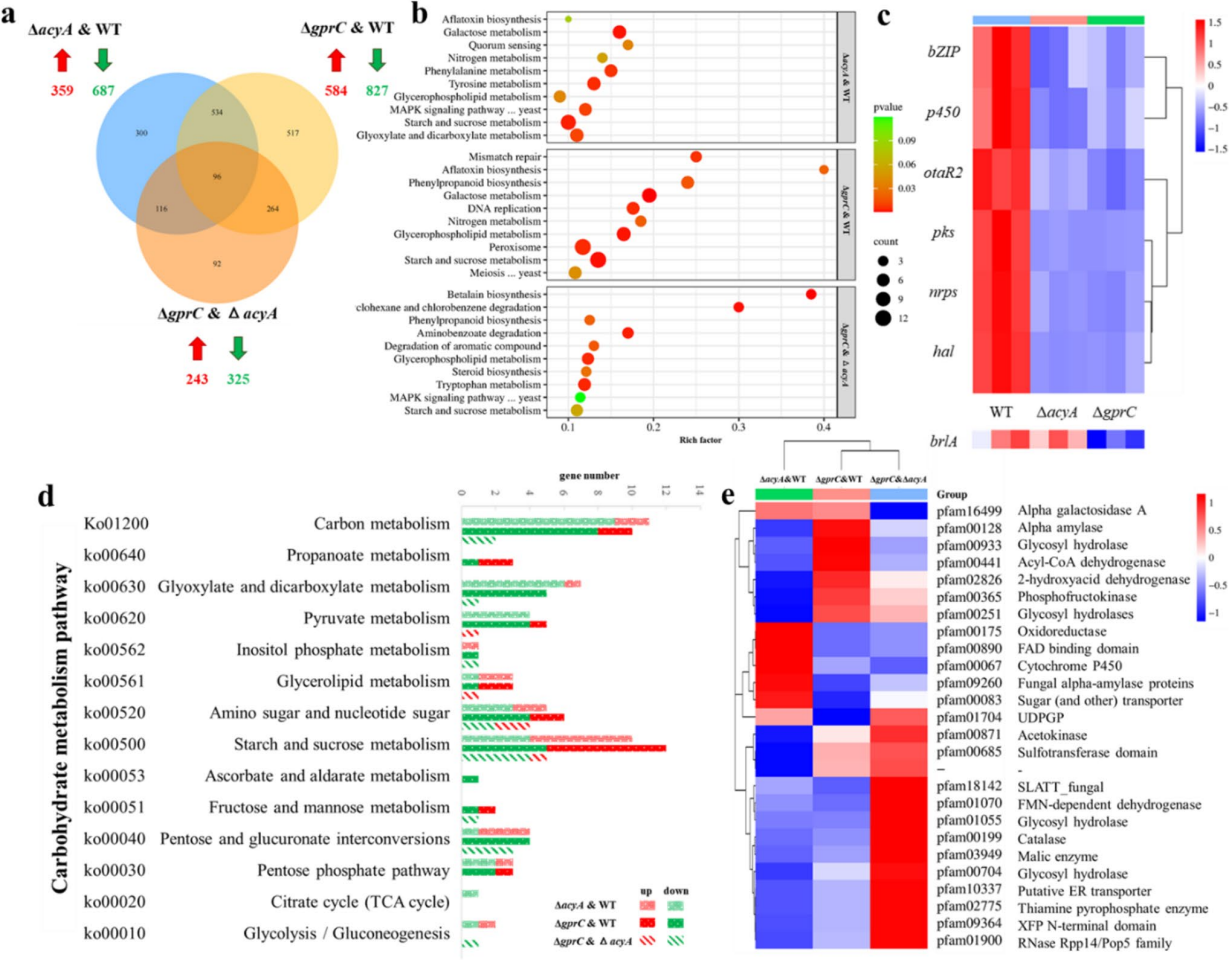
Transcriptomes pairwise aligned of wild type, Δ*gprC*, and Δ*acyA* after culturing 120 h in PDA. **a** Veen intersection of differentially expressed genes. **b** Bubble chart of representational enrichment pathways. **c** Gene expression heatmap of the OTA biosynthetic gene cluster that containing structural genes (*pks*, *cyc*, *nrps, p450*, *hal*), cluster-specific regulator (*bZIP*), and co-regulator (*otaR2*), as well as the central pathway that regulate sporulation, in which *brlA* is the only differently expressed gene. **d** DEGs statistics of KEGG carbohydrate metabolism pathways, and **e** heatmap of these DEGs

After screening out the GprC to AcyA-PKA pathway responsible for sensing and transmitting density information in wild type *A. ochraceus*, we knocked out the gene to investigate the functional mechanism of GprC and AcyA, which must be a complex regulatory process. Compared transcriptome perturbations among wild type, Δ*acyA*, Δ*gprC*, more genes were affected in Δ*gprC* than in Δ*acyA*, as well as Δ*gprC* still had 243 genes up-regulated and 325 genes down-regulated compared with Δ*acyA*, indicating there are parallel pathways from upstream GprC to downstream AcyA (Fig. 3a)*.* Pairwise compared mutants to WT showing the regulatory function of GprC to AcyA, signal transduction such as QS and MAPK were greatly affected, gene of the inhibited Gα subunit (Gαi) was down-regulated but the small G protein Ras was up-regulated, the down-regulation of p38 (a MAPK) affected the anti-hypertonic HOG pathway, and the 3-phosphoinositide dependent protein kinase-1 (PDK) function in phospholipase C (PLC) pathway was also down-regulated. In addition, GprC or AcyA deletion disrupted proliferation pathways, including meiosis, DNA replication, mismatch repair etc., and genes of the mini-chromosome maintenance (MCM) complex that controls the cell cycle were down-regulated (Fig. 3b, Figure S4 and Supplementary data 2).

Targeting OTA biosynthesis and spore production that is highly associated with the GprC-AcyA pathway, we found that they were regulated at the genetic level. Either GprC or AcyA deficiency limited the expression of all genes in OTA biosynthetic gene cluster (BGC), implying that their effect target is the cluster-specific transcription factor bZIP, and may also act on the coregulatory (OtaR2) [22]. For the BrlA-AbaA-WetA central regulatory pathway of sporulation, in which the regulatory factor BrlA was inhibited by PKA [23], deletion of AcyA slightly up-regulated whereas deletion of GprC significantly down-regulated *brlA* expression (Fig. 3c). This direct control of genes may explain why deletion of GprC or AcyA had fatal inhibitory effect on OTA biosynthesis and sporulation.

The nutrient competition will be enhanced with the increase of density, and the high density group of WT showed more active carbohydrate transport and metabolism (Fig. 2a), and either GprC or AcyA deletion disrupted carbohydrate metabolic pathways significantly (Fig. 3b). Genes related to gluconeogenesis, glycolysis, TCA cycle, and sugar transporter were down-regulated (Fig. 3d). In addition, extracellular carbon sources can be decomposed into monosaccharide by secreted polysaccharidase, the increased expression of polysaccharase genes in Δ*gprC* may due to the weakened glucose effect caused by glucose metabolism defects (Fig. 3e) indicates that GprC-AcyA participated in both extracellular carbon perception and intracellular carbon metabolism.

### GprC to AcyA mainly perceives glucose and acting on dormant spore germination

**Fig. 4:**
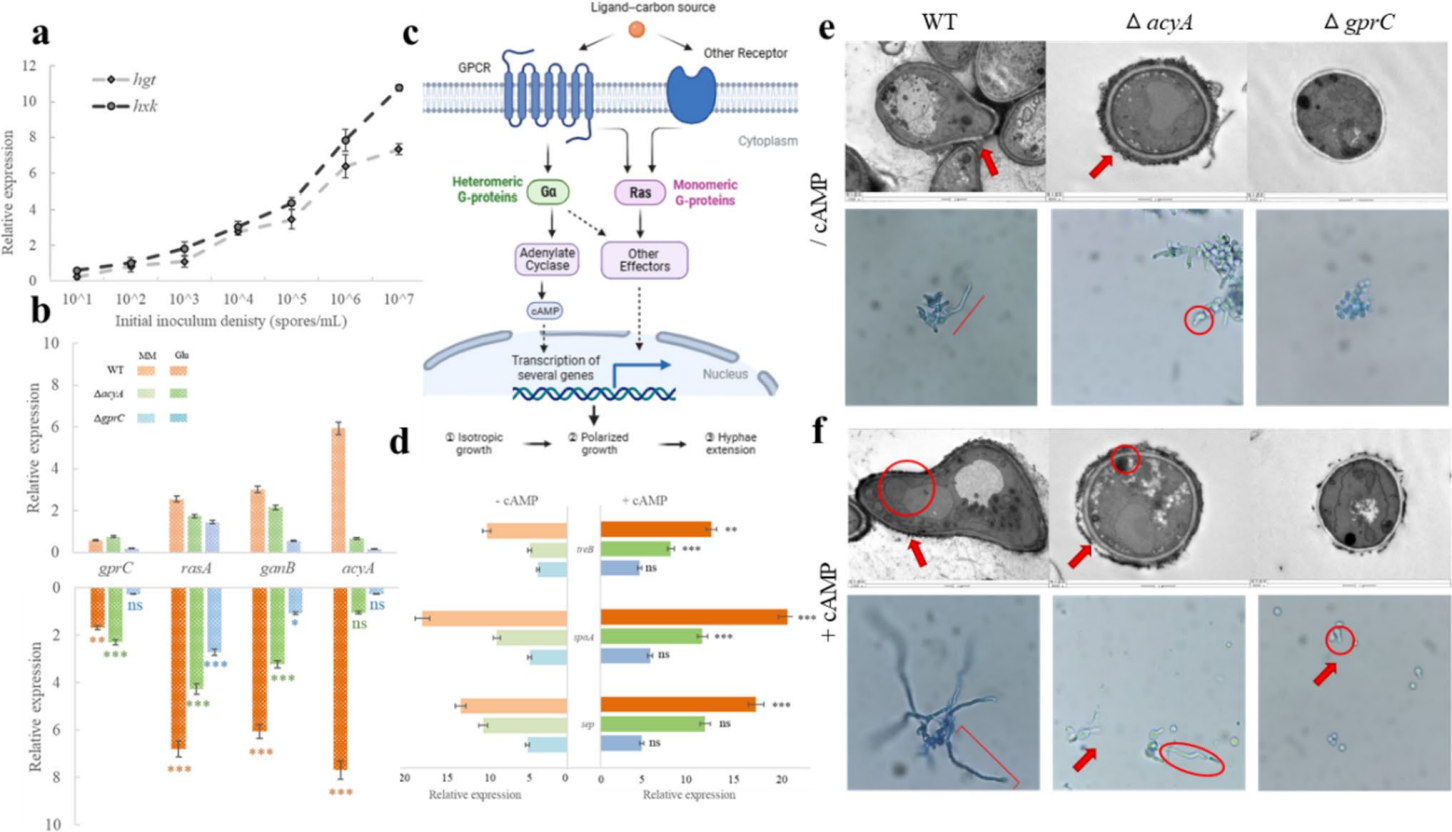
Effects of the GprC to AcyA pathway on glucose activating spore germination of *A. ochraceus*. **a** Density dependent transport and metabolism of glucose as the carbon source. **b** Relative expression of *gprC*, *rasA*, *ganB*, and *acyA* of WT, Δ*gprC* and Δ*acyA* in minimum medium (MM) and with glucose as the only carbon source. The (*) denotes the significant difference in gene expression between the same strain in MM and glucose medium, and different colors represent different strains. **c** G protein signaling pathways in germination. Glucose binding with membrane receptor to activate intracellular heterotrimer G protein (Gαβγ) or small G protein Ras, the Gα subunit-AcyA-cAMP pathway and Ras pathway are relatively independent, and affect spore germination by initiating gene transcription at different stages, including ①hypopus absorbency swelling and isotropic expansion at the early stage, ②bud tube germination at the middle stage, ③polarized extension at the late stage. **d** Spore germination related genes in three stages, including the trehalase encoding gene *TreB* that affects absorbency swelling during the isotropic growth phase, the polocyte forming gene *SpaA* that affects bud tube germination during the polar growth, and the septin forming gene *Spe* during the hyphae extension stage. The black (*) denotes the significance of the difference in gene expression in the same strain with or without cAMP supplementation. **e** Without or **f** with cAMP supplementation, the transmission electron microscopy (upper, magnification 4000) and light microscopy (bottom, magnification 400) observations of three strains

Glucose as the main carbon source and its transport and metabolism are enhanced with the increase of quorum density (Fig. 4a), indicating that extracellular glucose enters cells for utilization, which is consistent with the carbon metabolism, and is more active under high-density. Moreover, glucose is one of the necessary conditions to activate *Aspergillus* spore germination, but the gene expression and germination rate of *A. ochraceus* were higher under low-density (Fig. 1b). The regulation of GPCRs on spore germination has been globally studied, all GPCRs except class VIII GprO affected germination in *A. flavus* [16], whereas in *A. nidulans* [24] and *A. fumigatus* [25], only class III GprC and GprD were implicated in germination. In the studies of model fungus *A. nidulans*, Ras is primarily involved in water absorption to germ tube germination [26], and Gαs stimulates the AcyA-cAMP-PKA to regulate mycelial elongation [27, 28]. We hypothesize that glucose as the ligand binding with GprC to activate spore germination, which does not conflict with it as the carbon source during vegetative growth stage and occurs competitive utilization with increasing density, but the downstream effectors have not been explored.

In WT strain, glucose significantly activated the expression of receptor *gprC*, heterotrimeric G protein Gα subunit *ganB*, and effector *acyA*, deletion of AcyA did not affect the activation of glucose on GprC receptor and small G protein Ras, whereas Gα located upstream of AcyA would still be affected. In ∆*gprC*, the receptor deletion caused Gα and AcyA to no longer respond to glucose, but Ras expression remained up-regulated (Fig. 4b). This indicated that the activation order of glucose is (i) GprC to AcyA, (ii) GprC to Ras, which may also be triggered by other receptors. These two routes partially intersected and are relatively independent in *A. ochraceus* (Fig. 4c).

On the other hand, OTA was rarely produced during the germination stage (0–16 h) (Figure S2), suggesting that GprC acts on germination rather than OTA synthesis at this stage. Deletion of GprC and AcyA both reduced the expression of germination-related genes in *A. ochraceus*, including trehalase gene *TreB*, polar body formation gene *SpaA*, and septum formation gene *Sep* (Fig. 4d). Morphologically, in contrast to the enlargement of vacuoles and morphological change of WT spores activated by glucose, ∆*acyA* showed no apparent bud, while ∆*gprC* showed even no cell wall allosterism and swelling (Fig. 4e). Supplementing micromolecule cAMP to compensate the AcyA-PKA pathway, the expression of *treB* and *spaA* of ∆*acyA* could be compensated by cAMP, but *sep* that acting on the late stage of germination was not affected, and ∆*gprC* showed no respond to cAMP at all (Fig. 4d). Exogenous cAMP could promote septum formation and hyphae elongation in WT, and partially repair germ tube formation deficiency of ∆*acyA*, but it had little remedial effect on ∆*gprC* (Fig. 4f). Therefore, it was speculated that the transmembrane receptor GprC responses to glucose and activate spores to break dormancy, and the downstream AcyA-cAMP pathway via PKA primarily controls the middle and late stage of germination.

### GprC to AcyA pathway affects *A. ochraceus* development through regulating carbon metabolism

In *A. flavus* studies, Δ *A f gprC* mutant growth on several carbon sources was inhibited during the hyphal vegetative stage[16]. To assess the function of GprC-AcyA pathway on utilization of carbon source during *A. ochraceus* life cycle, we measured the spore germination, hyphal growth rate, colony stabilization, and secondary metabolism on four types of carbohydrate, including monosaccharide, disaccharide, polysaccharide, and alcohol.

**Fig. 5:**
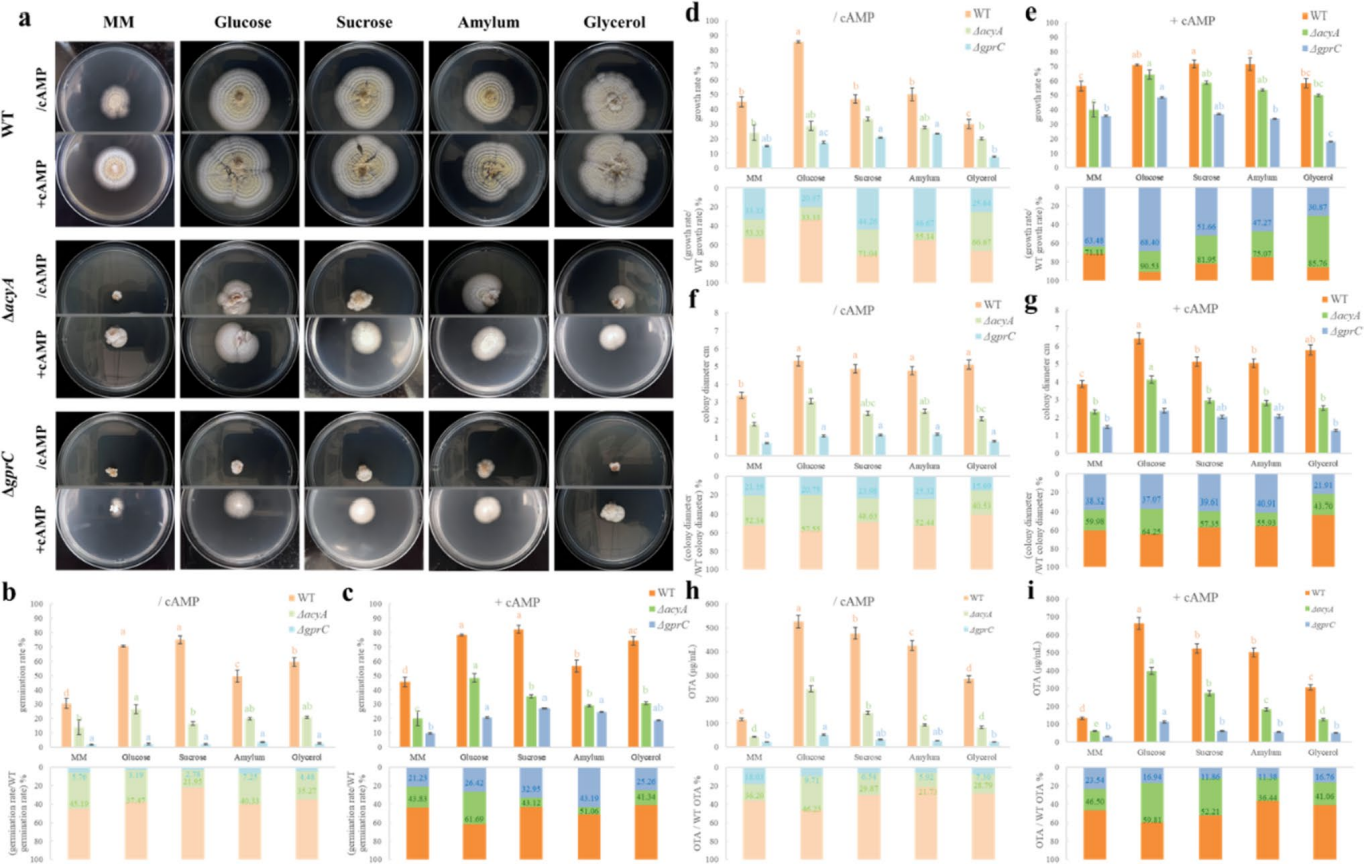
The role of G protein pathways in the utilization of carbon sources during the life cycle of *A. ochraceus*. **a** Final colony morphology of WT, Δ*acyA*, and Δ*gprC* in the absence and presence of supplementary cAMP. Without or with cAMP supplementation, the **b**, **c** germination rates at 14 h, **d**, **e** growth rates from 24 to 36 h, **f**, **g** stable colony diameter after culturing 120 h, **h**, **i** OTA production after culturing 120 h. Colored lowercase letters indicate the significance of differences in growth of the same strain in different carbon sources, *P* < 0.05

Mutants Δ*gprC* and Δ*acyA* preform significant phenotypic difference from the wild type (Fig. 5a). The germination rate of *ΔacyA* decreased significantly and was no longer stimulated except for glucose, while the Δ*gprC* was nearly entirely restricted in any carbon source (Fig. 5b). Supplement of cAMP could compensate partly, the germination rate of Δ*acyA* in glucose was drastically restored from 37.85% to 62.29% of WT, and Δ*gprC* was restored from less than 10% to 20 ~ 40% of WT. There is no significant difference between the activation of mutant spore germination by different carbon sources, may because the intracellular effector was activated to the same degree of cAMP and no longer involved receptor preference for carbon sources (Fig. 5c). When glucose was the only carbon source, the growth rate of WT was significantly faster than Δ*gprC* and Δ*acyA* (Fig. 5d). After cAMP supplementation, mutants’ growth rate were recovered even in the absence of carbon supply (in minimal medium), particularly in glucose where Δ*acyA* recovered from 33.33% to 90.53% of WT, and Δ*gprC* recovered from 20.37% to 68.40% of WT (Fig. 5e). Colony diameters of each strain were equivalent in four carbon sources (Fig. 5f), adding cAMP could partially compensate for the mycelial elongation defect but mutants remained significantly smaller than WT (Fig. 5g). Finally, OTA produced by WT, Δ*acyA*, and Δ*gprC* in four carbon sources were different but all significantly more than in MM (Fig. 5h). Such differences still existed even after AMP supplementation, while the OTA production could be recovered by cAMP in mutant strains (Fig. 5i).

### GprC-AcyA pathway responsible for transmitting HODEs-mediated intraspecific and interspecific communication

Oxylipins are produced inside the cell but secreted outside to perform its QSM function, and affecting the density dependent production of OTA and spore in *A. ochraceus* [29]. However, the composition of autocrine QSM and the response mechanism have not been analyzed in *Aspergillus*. In addition, the same oxylipin produced by plant whether contributing in fungi-plant infection systems, which may relaxed to the fungal preference of hosts. We screened HODEs and prostaglandin E_2_ (PGE_2_) that may carry quorum density information by metabolomics [29] and wanted to analyze the transmission of GprC-AcyA pathway in both intra-fungus and fungus-plant system.

**Fig. 6:**
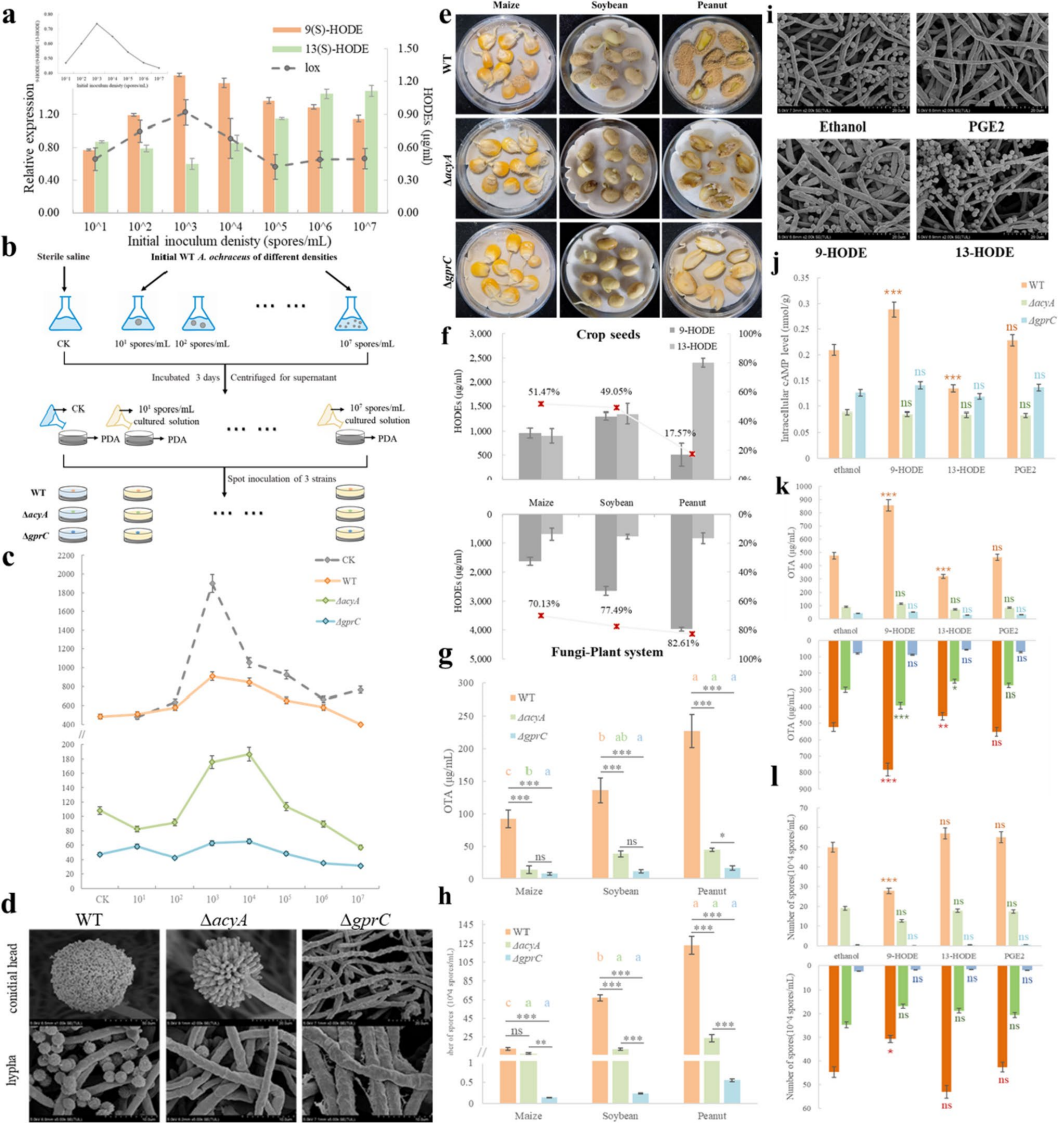
GprC-AcyA pathway transmit intra- and interspecific communication signals as well as the effect of standard oxylipins on *A. ochraceus*. **a** The concentration of HODEs (histogram), expression of *lox* (dotted line), and proportion of 9-HODE (embedded line graph) of WT in serial density systems after culturing 72 h in PDB. **b** Experimental flow diagram of the effect of autocrine signaling molecule systems. **c** Effect of autocrine signaling molecules on OTA production in WT, Δ*acyA*, and Δ*gprC*. **d** Scanning electron microscopy of the asexual propagation structure of three strains, each scale indicates 50 μm (upper) and 10 μm (bottom). **e** The varying degrees of three strains infect maize, soybean, and peanut seeds. **f** The concentration of HODEs (histogram) and proportion of 9-HODE (line graph) in crop seeds (upper) and WT *A. ochraceus*-plant infection system (bottom) after culturing 72 h. The **g** OTA production and **h** spore quantity in fungi-plant infection system after culturing 72 h. The black asterisk represents the difference between three strains in the same seed, the colored letters represent the differences of the same strain in three seeds, different colors indicate different strains. **i** Scanning electron microscopy of WT affected by PGE_2_, 9-HODE, and 13-HODE. **j** intracellular cAMP levels of three strains affected by standard oxylipins. **k** OTA biosynthesis and **l** spore generation with or without additional cAMP in WT, Δ*acyA*, and Δ*gprC*. The colored asterisk denotes the significance of difference in response to different oxylipins in the same strain compared to the ethanol control (*) *P* < 0.05, (**) *P* < 0.01, (***) *P* < 0.001, (ns) non-significant

In the intraspecific QS culture system, the concentration of 9-HODE as well as proportion of 9-HODE in HODEs were consistent with OTA that also peaked at the 10^3^ spores/mL, while the 13-HODE was like the trend of spore number. The expression of *lox*, gene encoding the oxidase 9-LOX that catalyzes the oxidation of linoleic acid to produce 9-HODE, was similarly density-dependent, associated with "fungi quroum density—quorum sensing molecule (9-HODE)—OTA” (Fig. 6a). Then we re-inoculated new WT, Δ*gprC* and Δ*acyA* mycelium on the supernatant culture solution of WT *A. ochraceus*, containing fungi autocrine oxylipin systems (Fig. 6b). WT still produced the most OTA in the 10^3^ spores/mL culture medium. As Δ*acyA*, the OTA production reduced significantly and the largest yield was in a higher density culture medium (10^4^ spores/mL), while the OTA production of Δ*gprC* was completely inhibited and did not respond to any systems (Fig. 6c). For the sporulation, WT strain hyphae were smooth and normally differentiated into thick conidiophore stipes, metula, phialide, and meiosis to produce clusters of conidia. In Δ*acyA*, mycelia continue to differentiate but the spore head diameter is only about 40% of the normal one, and phialide asymmetric division appears to be blocked. In Δ*gprC*, hyphae got thicker and more wrinkled; no hypha differentiated into foot cells and no spores were observed (Fig. 6d).

In the fungi–plant interaction system, fungal coverage was peanut** > **soybean** > **maize in WT strain, which increased with the intrinsic lipid content of crop seeds (Figure S5). The deletion of GprC and AcyA both impaired the infection ability of *A. ochraceus* (Fig. 6e). Among these three most contaminated grains with different nutritional components, their own oxylipin composition are different and the 9-HODE proportion of plant hosts increased significantly during the *A. ochraceus* infection. Peanuts with the most total lipid but the least 9-HODE were the most susceptible, while 9-HODE rose to 82% after interacting with fungi (Fig. 6f), speculating related to resistance mechanisms of both sides. For OTA production, Δ*acyA* and Δ*gprC* had nearly the same degree of defects, except for a significant difference in peanut that containing the most lipids (Fig. 6g). For sporulation, mutants are no longer affected by hosts and there is no difference in three seeds, Δ*acyA* spores are consistently significantly more than Δ*gprC* and are not different with WT in maize that contain the fewest lipids (Fig. 6h).

Finally, to exclude the influence of other limiting factors such as nutrient sources and functional molecules and target oxylipins, we added oxylipin standards in base medium and determined key indicators (Fig. 6i). In WT, 9-HODE promoted but 13-HODE inhibited the cAMP level, while PGE_2_ had no effect (Fig. 6j), suggesting that these molecules may be the signal that can activate AcyA-PKA pathway then promote OTA biosynthesis while inhibit sporulation. However, Δ*acyA* that lacks the only cAMP catalytic enzyme, and Δ*gprC* that lacks the signaling receptor, no longer produce cAMP that in response to HODEs, and OTA or spore were unaffected. After cAMP supplementation, the responsiveness of OTA biosynthesis to HODEs was partially restored in Δ*acyA*, but Δ*gprC* remained unresponsive, indicating that the cAMP production defect was compensated, while the ligand perception defect could not be restored by the supplement of downstream second messenger (Fig. 6k, l), suggesting HODEs as potential ligand for GPCR in the stimulation of OTA synthesis and inhibition of asexual spore production.

## Discussion

In *Aspergillus* spp*.*, the relationship between density and behavior that shown as quorum sensing has been studied for a long time. Studies of *A. nidulans* [30], *A. parasiticus* [31], *A. flavus* [32], and *A. ochraceus* [33] have found that morphological transformation and secondary metabolites are regulated in a density-dependent manner mediated by quorum sensing molecules. However, there are inconformity between existing results and the density threshold remains uncertain, which are due to differences in filamentous fungus properties and experimental conditions, but there may be more influencing factors that have not been studied. Therefore, we first explored the mechanism of QS in *A. ochraceus*, which has strong ability to produce OTA, hoping to provide some complement for fungi cell communication.

*Aspergillus ochraceus* behaviors are density-dependent, and shift after quorum density reaching the threshold (10^3^ spores/mL). Transcriptome comparison showed that fungal emphasis under different density conditions was changed, which was mainly reflected in that secondary metabolism was more active under low-density, while primary carbon metabolism was more active under high-density. In addition, lipids metabolism and transport system were always important, which were related to the synthesis of oxylipins catalyzed by oxidases and the transport of signals via phospholipid bilayer membrane. The intracellular AcyA-PKA pathway seemed to be mainly responsible for the information transport. What is more important is the perception by membrane receptors, the GPCR family can sense a diversity of signaling molecules, among which GprC may be one of the response density information. We have systematically performed sequence similarity comparison and phylogenetic evolution analysis of the *A. ochraceus* GPCR family, and GprC was classified as the glucose sensor according to homology [34]. The molecular docking showing that structural constraint of GprC binding with ligands, glucose and 9-HODE, are not really strict, which may explain how various signals activate cells to perform relevant functions at different life cycle stages. In addition, resolving the crystal structure of ligand and receptor can be a more intuitive method, and it is also necessary for further study. According to this, specific structural recognition can find more possible ligands, as well as develop activators or inhibitors against *Aspergillus* spp.

**Fig. 7:**
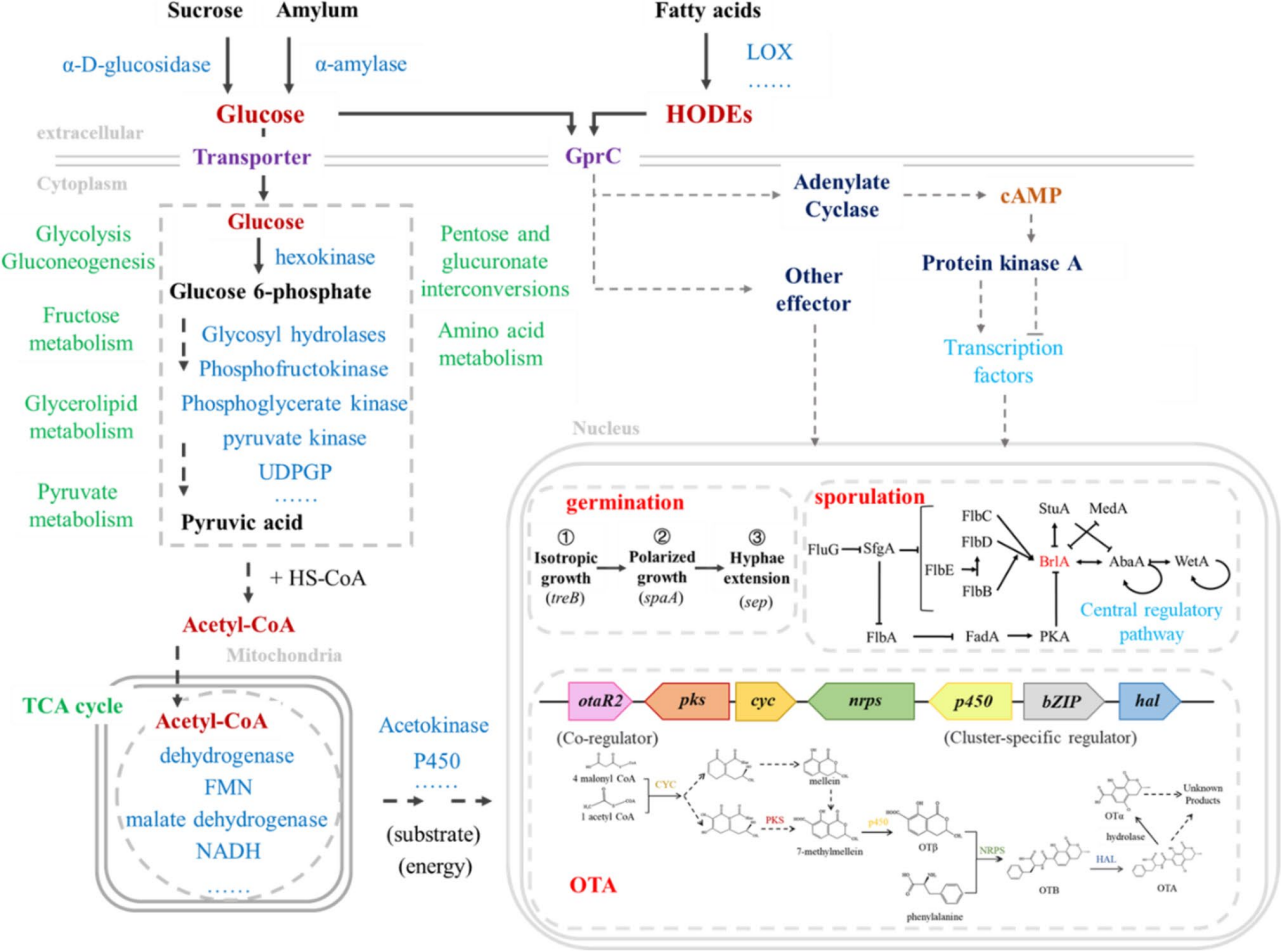
The hypothesized comprehensive mechanism of carbon source as well as QSM influence on *A. ochraceus* behaviours

In fact, population density causes fungal adaptive behavioral changes primarily through nutritional restriction. Glucose is an important nutrient source that can cross into the cell through membrane transporter, after a series of metabolic processes such as the tricarboxylic acid cycle, providing substrate and energy for fungal development. Compared to polysaccharides that must first be degraded by secreted enzymes, glucose has a lower metabolic cost [35], and its metabolites will inhibit the cAMP-activated production of polysaccharases, achieving glucose preferential utilization in the presence of multiple carbons, which is known as the glucose effect [36]. However, besides being transported into cells and as the substrate to be metabolized, we verified that extracellular glucose also can activate adenylate cyclase to produce cAMP by binding with GPCRs as the ligand, and then regulates middle and late stages of spore germination, as well as affects various carbon metabolism during mycelial vegetative growth. In conclusion, as the ligand for GprC, glucose can activate carbohydrate metabolism and OTA production during hyphae growth, moreover, as the metabolic carbon source, glucose can supply energy for fungal growth and substrate acetyl-CoA for OTA biosynthesis, reflecting the direct and indirect regulation of OTA biosynthesis by GprC-AcyA pathway (Fig. 7).

In addition, the GprC-AcyA pathway has a negative feedback on glucose effect, as well as competitive glucose utilization also regulates secondary metabolism and spore production besides affected by cell communication molecule QSM. These may explain why *Aspergillus* exhibiting inconsistent density-dependent behavioral shifts. At present, the main QSM identified in fungus including alcohols, small peptides, aldehydes and volatile organic compounds, among which oxylipins attracting the most attention. They are universal communication signals among microorganisms, plants, and animals based on homologous oxidases and unsaturated fatty acids substrates [37, 38]. Among oxylipin, PGE_2_ as an animals immune factor binding with GPCR, and also produced in *A. fumigatus* [39] but exogenously added did not act on *A. ochraceus* in our work. HODEs can be produced by crops but function on fungus, and vice versa [40]. For example, fungus-derived 9-HODE and HETEs can cause programmed cell death in tomato protoplasts [41]. Tomato and corn can produce 9-HPODE that promotes aflatoxin (AF) synthesis, while soybean and peanut can produce 13-HPODE promotes sporulation but inhibits AF [42]. 5,8-diHODE and 7,8-diHODE induce cell differentiation and lateral branching, as well as regulating sexual development and sporulation in *Aspergillus* [43]. We found in *A. ochraceu*s that likely because 9-/13-HODE may recognized by GprC to activate Gαs/Gαi subunits, respectively, then activating or inhibiting AcyA to catalyze synthesis cAMP. The cAMP effector protein PKA can promote OTA biosynthesis through bZIP, and inhibit spore formation through BrlA, thus shown that 9-HODE promoted OTA and inhibited spores production, and 13-HODE inhibited OTA, these results are consistent with phenomena shown in *A. nidulans* and *A. flavus* [10, 44]. In conclusion, we deduce a possible way of responding to QSM, which is the “HODEs–GprC–AcyA-cAMP-PKA–regulatory factors–genes” cascade.

In conclusion, both GprC and AcyA are pivotal links that located upstream and downstream of a complex but efficient G protein network, which totally regulates fungal whole life cycle. Contributing to individual microorganisms withstanding comprehensive environmental stresses, as well as facilitating adaptive communication for co-evolution across species in the same ecosystem [40]. However, the growth and metabolic defects caused by deleting the upstream or downstream components in this pathway were distinct, only interfered cellular communication theoretically not lethal. Suggesting that GprC and AcyA may have additional functions besides as hubs of environmental signal transmission, which requires further study.

**Fig. 8:**
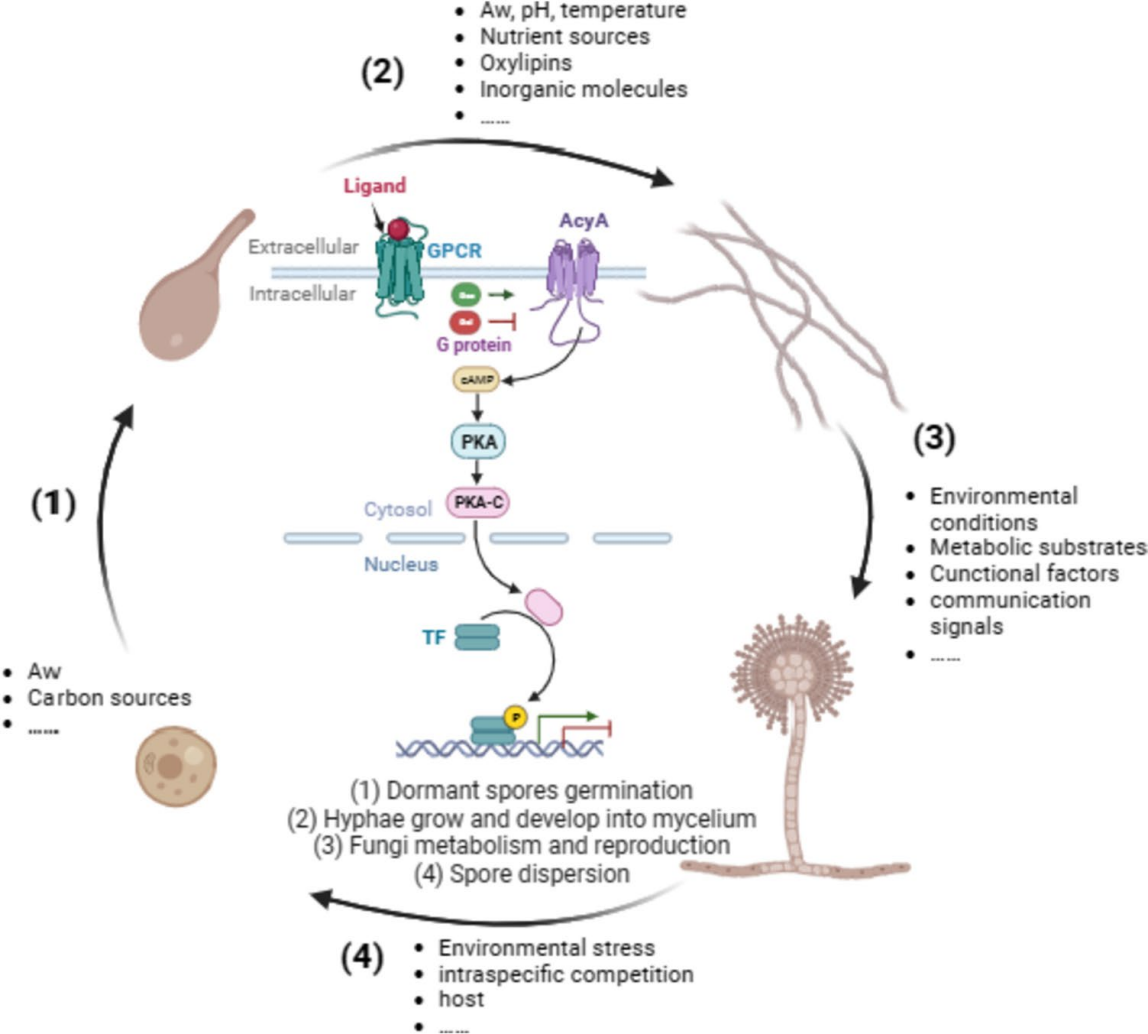
The GprC-AcyA pathway senses a wide range of signals and regulates the life cycle of *Aspergillus*. (1, 2, 3, 4) representing different stages of life cycle, facing environmental stresses, and the G protein pathway, consisting of ligand, receptor, effector, second messenger, and regulatory factors, can respond to various cues and achieve adaptive regulation of behavior

Although this article concentrated on the “HODEs (QSM/first messenger/ligand)—GprC (receptor)—AcyA-cAMP (second messenger)-PKA—regulatory factors—genes—behavioral shifts” cascade pathway in *A. ochraceus*, we predicted that more analogous GPCR cascades may exist in broader *Aspergillus* genus and play more functions (Fig. 8). In the future, ligands, receptors, and effectors are all potential targets for artificial intervention. Flexible regulations focus on microbial behaviors controlled by signaling transmission, such as quorum quencher (QQ) can prevent fungal contamination and pathogenesis [45]. On the other hand, the stimulation of potential gene by activators or inhibitors can be used to develop cell factories [46].

## Methods

### Chemicals and reagents

OTA standard (purity ≥ 99%) was purchased from Fermentek (Jerusalem, Israel). cAMP standard (purity ≥ 98%) was purchased from Solar Bio (Beijing China). Oxylipins 9(S)-HODE (purity ≥ 98%), 13(S)-HODE (purity ≥ 98%), and PGE_2_ (purity ≥ 98%) were purchased from Cayman Chemical (Michigan, USA). The stock solutions were dissolved in ethanol and stored at − 80 °C, and then diluted with ethanol to working solutions before analysis. Other conventional solvents used in this experiment are chromatographic purity, all purchased from Merck (Darmstadt, Germany).

### Fungal strains

*Aspergillus ochraceus* wild-type strain (CGMCC No. 3.4412) was purchased from the Institute of Microbiology, Chinese Academy of Sciences and preserved in glycerol at − 80 °C in lab. This strain derived from the original discovery of mycotoxigenic strains fc-1 (NCBI BioSample ID: SAMN03140103). A non-homologous end joining (NHEJ) defect that has highly efficient homologous recombinant (HR) strain Ao*ku70* (gene type: ΔAo*pyrG* ΔAo*ku70*::Ao*pyrG*) was used for mutation and was purchased from the Chinese Academy of Agricultural Sciences. *Aspergillus* strains were activated on the Potato Dextrose Agar (PDA) medium at 28 °C for 7 days in the dark and repeated passage three times.

### Mutant strains construction

GprC and AcyA deletion strains were constructed by homologous recombination, to interrupt ligands recognition and intracellular signal transmission mediated by cAMP, respectively. Upstream and downstream regions of the respective genes with a length of 1.0 kb were amplified from the genomic DNA of *A. ochraceus* and the *hygB* gene was amplified from plasmid pCAMBIA1300 as a selective marker. Double-joint fusion PCR products (*gprC* and *acyA* gene deletion cassette) were transformed into the protoplasts of *A. ochraceus* by polyethylene glycol–mediated protoplast transformation according to the method described previously [47]. After incubation at 28 °C for 3 days, transformants were transferred onto fresh screening plates prepared with 0.8 mol/L sorbitol and containing hygromycin B (100 µg/mL) for second selection. The Screening of *ΔgprC* and Δ*acyA* transformants was carried out by diagnostic PCR amplification (Figure S7) and oligo-nucleotide sequences for PCR primers are given in table S1.

### Culture conditions

Minimal medium (MM) that supported with 1% (w/v) different types of carbon (monosaccharide–glucose, disaccharide–sucrose, polysaccharide–amylium, and alcohol–glycerol) were used to study the utilization of carbon sources by *A. ochraceus*.

To assess the effects of specific oxylipins, 9-HODE, 13-HODE and PGE_2_ standards were added to PDA medium at a final concentration of 5 μmol/mL, which determined by the effect of exogenous oxylipins (Figure S6) and the same volume of ethanol was used as the solvent control.

Signaling molecular medium were used to analyze the effects of endogenous oxylipin systems on *A. ochraceus*. A series densities of WT strains were inoculated in potato dextrose broth (PDB) liquid medium to reach final densities of 10^1^ ~ 10^7^ spores/mL, and the same volume of sterile saline as the blank control, all cultures were incubated at 28 °C for 3 days in the dark. Centrifuged supernatants, containing the self-secreted signaling molecules, was added with agar at the final concentration of 1% separately. After homogenization and autoclaving, 5 ml of the supernatant was poured on the surface of the prepared PDA solid medium, and was used to culture Δ*gprC*, Δ*acyA* and WT strains after cooling and solidification.

### qRT-PCR analysis

cDNA was prepared using TransScript® All-in-One First-Strand cDNA Synthesis SuperMix (TransGen Biotech, Beijing, China). qRT-PCR was performed with the Thermo Fisher Scientific Real-time PCR System using TransStart Top Green qPCR SuperMix (TransGen Biotech, Beijing, China), and the primers were listed in Table S1. The PCR amplification program was developed by Gao [22].

### Transcriptome analysis

Total RNAs of each samples were extracted with TransZol Up Plus RNA Kit (TransGen Biotech, Beijing, China). RNA concentration and purity was measured using NanoDrop 2000. RNA integrity was assessed using the RNA Nano 6000 Assay Kit. Sequencing libraries were generated using NEBNext®Ultra™ RNA Library Prep Kit for Illumina® (NEB, USA). The sequences were further processed with BMKCloud (www.biocloud.net) online platform. Gene expression levels were estimated by cDNA fragments per kilobase of transcript per million fragments mapped. Differential expression analysis of two groups was performed using the DESeq2. Genes with an adjusted p value < 0.05 found by DESeq2 were assigned as differentially expressed. The cluster analysis was conducted by blasting with gene ontology (GO) and Kyoto Encyclopedia of Genes and Genomes (KEGG). Functional enrichment analysis (GO and KEGG pathway) was conducted with KOBAS software.

### Sporulation and spore germination rate assays

Asexual spore inoculating solution was prepared after culturing on solid mediums for 5 days, washed spores by sterile saline containing 0.05% Tween 80 and counted it using a hemacytometer under the light microscope, the final concentration of spore suspension was adjusted to 10^8^ spores/mL and then diluted when necessary. Quantitative sporulation by punching a 1 cm diameter solid medium with spores then resuspension and count, each count was repeated three times parallel.

Spore suspension of 10^5^ spores/mL was cultured in liquid medium at 28 ℃ in dark for 16 h, and every 2 h to observe germination under the light microscope (Figure S1). The bud tube longer than the spore radius can be considered successful germination, and the calculation formula is: Germination rate (%) = number of spores germination/total number of spores × 100.

### Mycelial growth and phenotypic assays/observation

The growth curve of *Aspergillus ochraceus* was plotted in advance and the trend of OTA production with culture time was measured (Figure S2). To evaluate the various influence on fungal mycelial growth, *A. ochraceus* strains were spot-inoculated on the center of different solid mediums and incubated at 28 °C in dark. The mycelial diameters were recorded every 12 h until 120 h, and the mycelial growth rate was calculated by the following formula: growth rate (%) = (T2 − T1/T1) × 100%, where T1 is the mean colony diameter at a certain time and T2 is the respective value after 12 h.

To observe the mycelium tissue or spores surface, the 48-h-old *A. ochraceus* were collected from solid medium and fixed with 2.5% glutaraldehyde at 4 °C overnight, then washed with PBS three times and dehydrated in ethanol, critical point dried, and finally sputter coated with gold for the scanning electron microscope (SEM) observation.

To observe the changes of intracellular organelles and nuclei, germinating cells grown for 14 h in liquid medium were fixed in 2.5% glutaraldehyde for 3 h and washed with PBS three times, then post fixed in 1.0% osmium tetroxide for 2 h and dehydrated in graded ethanol series (30–100%). The blocks were sectioned, stained, and observed using a transmission electron microscopy (TEM).

### OTA extraction and detection

The OTA of all systems was extracted and detected using methods developed by Shi et al. [48]. Briefly, all samples were acidified with 2 mol/L HCl and extracted twice with CHCl_3_. After ultrasonic vibrating for 30 min and vortexing for 3 min, OTA was recovered by centrifugation of the extracts at 8000 rpm for 5 min at 4 °C. The CHCl_3_ extracts were evaporated, re-dissolved with methanol, filtered using 0.22 μm cellulose pyrogen-free filters, and used for HPLC analysis.

The OTA was detected on an Agilent high-performance liquid chromatography (HPLC) system coupled with a fluorescence detector (FD) at excitation and emission wavelengths of 334 and 460 nm, respectively. OTA was eluted through C18 column (250 mm × 4 mm, particle size 10 μm) at a flow rate of 1 mL/min of mobile phase (acetonitrile: water: acetic acid = 99:99:2, V:V:V).

### cAMP extraction and detection

The intracellular cAMP of *A. ochraceus* was extracted and detected using methods developed by Affeldt et al. [44] Briefly, at least 10 mg freeze-dried mycelium samples were homogenized in 100 μL lysis buffer, and then incubated for 10 min on ice and centrifuged at 13,000 rpm for 5 min at 4 °C.

cAMP was measured using the Direct cAMP ELISA Kit (Elabscience Biotechnology Co., Ltd, China) according to the manufacturer’s directions. Reactions were performed in 96 well plates and optical density (OD450) was measured using the Infinite® F50 Plus multiwell plate reader.

### HODEs extraction and detection

The intracellular 9-HODE and 13-HODE of *A. ochraceus* were extracted and detected using HPLC–MS/MS method that developed by Gao et al. [49]. Briefly, all samples were vacuum freeze-dried for 48 h to terminate the metabolism, and ultrasonic extraction was performed for 1 h using chloroform: methanol = 2:1 (V: V) mixed extractant. HODEs were recovered by centrifugation of the extracts at 8000 rpm for 5 min at 4 °C. The organic phase extracts were evaporated, re-dissolved with methanol, filtered using 0.22 μm cellulose pyrogen-free filters, and used for HPLC–MS analysis.

HPLC used isocratic elution and the mobile phase was acetonitrile: 0.1% formic acid = 90:10 (V:V), the flow rate was 0.4 mL/min, the injection volume was 10 μL. HODEs were eluted through Eclipse plus C18 column (2.1 mm × 100 mm, particle size 3.5 μm) with a column temperature of 30 °C. Mass spectrometry conditions were as following: electrospray ion source negative ion (ESI) mode, the ion source temperature was 100 °C, the drying gas temperature was 350 °C and flow rate was 8 min, the atomizing gas pressure was 35.0 psi. The fragmentation voltage is 135 V and the multiplex reaction monitoring (MRM) mode was selected for the detection method. At specific retention time intervals, the intensity of the characteristic daughter ions in the MS2 scan for 9-HODE (m/z = 57/296) and 13-HODE (m/z = 81/296).

### Seed infections

The surface of dried seed of peanut, soybean, and maize were disinfected with 0.05% sodium hypochlorite for 3 min, 70% ethanol for 3 min and rinsed with sterilized water for 30 s three times. Sterilized seeds were immersed in sterile distilled water (control) or sterile distilled water with fungal conidia suspensions of different densities in flasks while shaking 30 min at 120 rpm under 28 °C for infection. This was followed by complete drainage, then the seeds after infection were placed in 60 mm disposable petri dish with three layers of sterile filter paper that moistened with 2 ml sterile water to maintain high humidity. All steps were performed aseptically in a biosafety hood and incubated at 28 ℃ in the dark for 7 days. Add an additional 1 ml of sterile distilled water to each dish daily to maintain appropriate moisture levels during the experiment.

### Molecular docking

The 3D structure of the receptor protein was predicted by AlphaFold 2 software, The sdf structure of small molecules were obtained from the Pubchem database and converted into PDB files by OpenBabel. AutoDock Tools 1.5.6 was used to dehydrate and hydrogenate the protein target, and AutoDock Vina software was then used for molecular docking. Finally, the results with strong binding force were selected and visualized using Maestro 11.5 software.

### Statistical analysis

All data are expressed as mean value ± standard error, calculated from three or more independent replicates. For comparisons between two treatment groups, the Welch’s two-sided t test was performed. Comparisons across multiple conditions or strains were performed using Brown–Forsythe and Welch ANOVA tests followed by Dunnett’s T3 multiple comparison test. Comparisons of two strains with two or more conditions were performed via two-way ANOVA test followed the Holm–Šídák multiple comparison test to compare between strains within each treatment. Comparisons of multiple strains grown in the same condition were made using one-way ANOVA followed by Dunnett’s multiple comparison tests. All analyses used a cut-off P value = 0.05 for statistical significance.

### Supplementary Information

Below is the link to the electronic supplementary material.

Supplementary file1 (DOCX 1903 KB)

## Data Availability

All authors have approved the availability of data and materials in the manuscript and agree with its submission to Cellular and Molecular Life Sciences.
