# Supplementary material for: Glucose and HODEs regulate Aspergillus ochraceus quorum sensing through the GprC-AcyA pathway
Source: Cell Mol Life Sci. 2024 May 29;81(1):241. doi: 10.1007/s00018-024-05160-z (PMC11133280; doi:10.1007/s00018-024-05160-z)
Supplement: Supplementary file 1 — Supplementary file1 (DOCX 1903 KB) [file 18_2024_5160_MOESM1_ESM.docx]

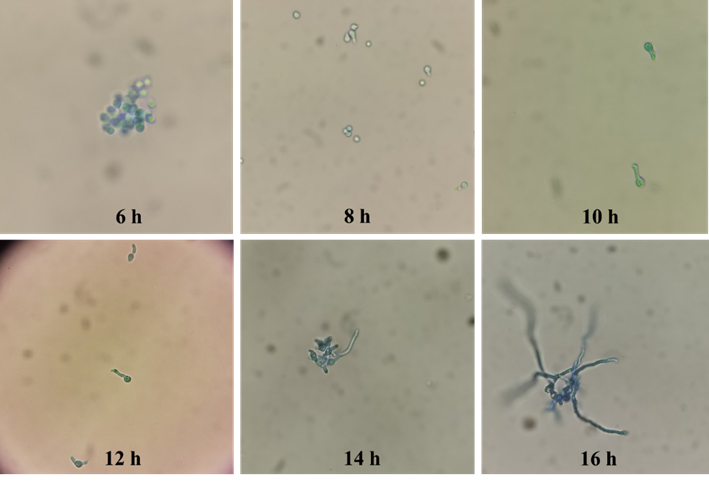


**Figure S1** Observation of spores germination. The magnification of the optical microscope is 400×. Spores inoculated in liquid medium were observed for germination every 2 hours，when the bud tube longer than the spore radius can be considered successful germination


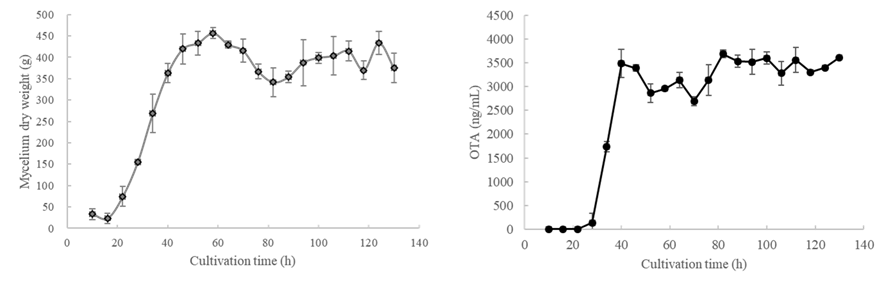


**Figure S2** Changes in mycelial dry weight (growth curve) and OTA production with culture time. Using the dry weight of mycelium as the main index, and the OTA production was measured at the same time, sampling every of 6 h, and culture under constant conditions for a total of 144 hours, which could cover the complete growth cycle of *Aspergillus ochraceus*.


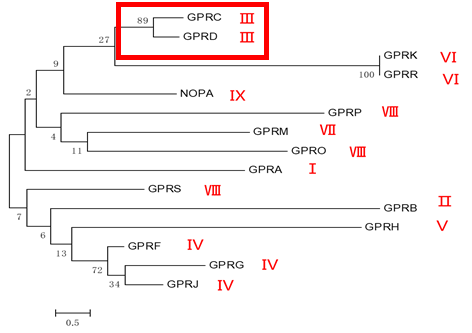


**Figure S3** Phylogenetic cluster analysis of Aspergillus ochracceus GPCR family


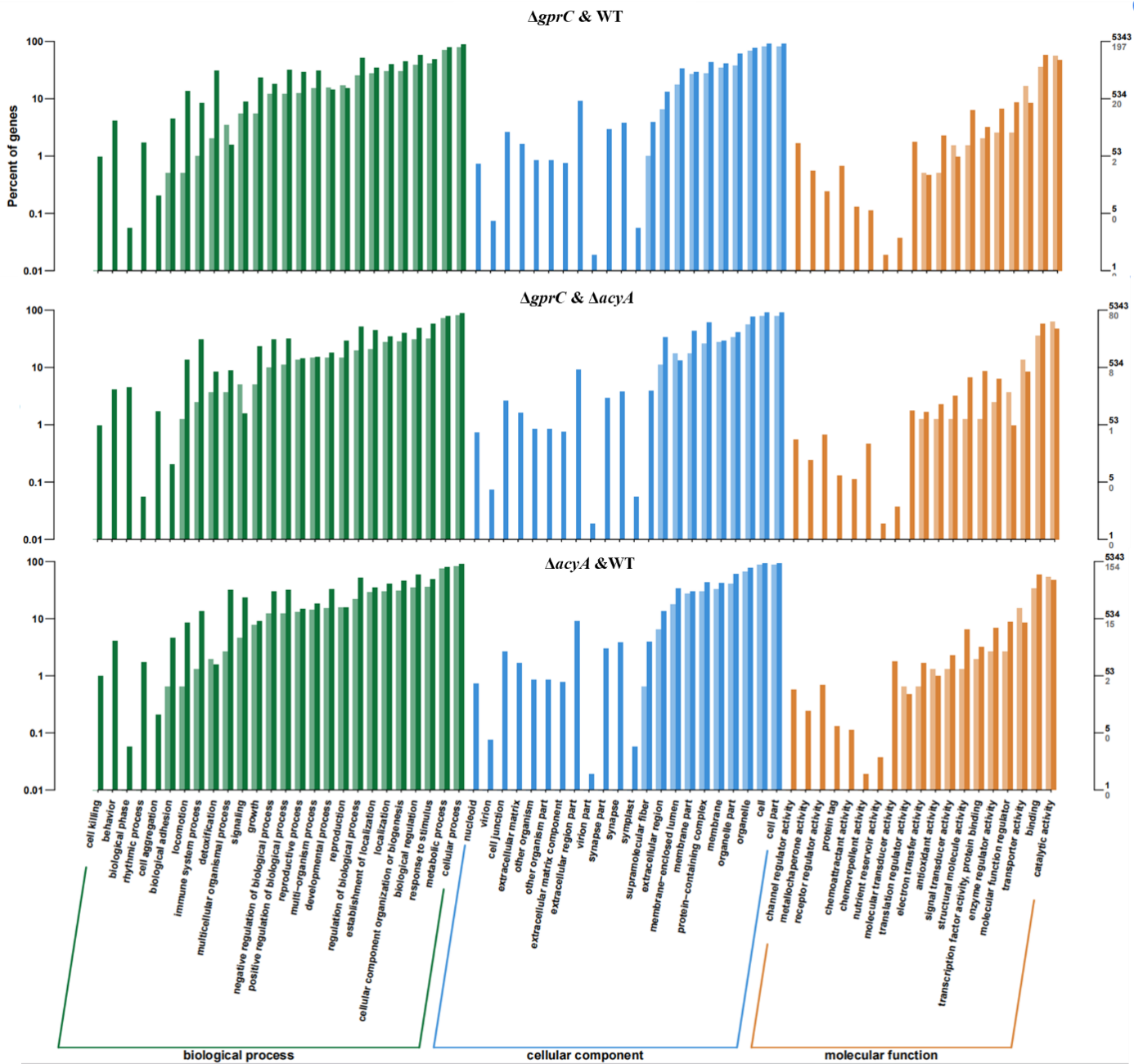


**Figure S4a** Transcriptomes analysis of wild type, Δ*gprC*, and Δ*acyA*. KOG enrichment of differentially expressed genes (DEGs).


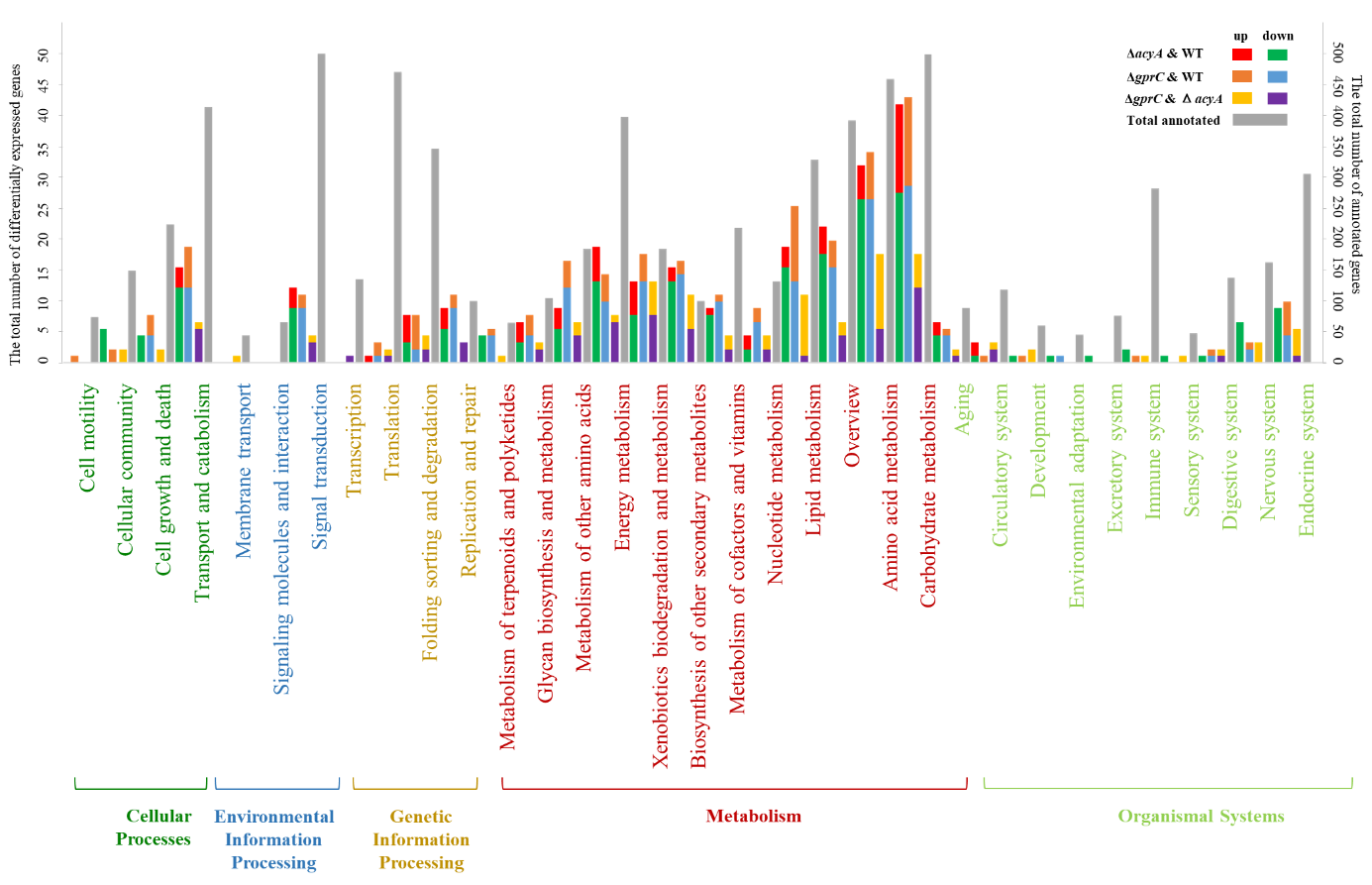


**Figure S4b** Transcriptomes analysis of wild type, Δ*gprC*, and Δ*acyA*. KEGG enrichment of differentially expressed genes (DEGs).

|  | Fat (g/100g) | Protein (g/100g) | Carbohydrates (g/100g) |
| --- | --- | --- | --- |
| Peanut | 44.8 | 22.1 | 23.8 |
| Soybean | 18.5 | 29.6 | 15.4 |
| Maize | 1 | 8.2 | 76.4 |


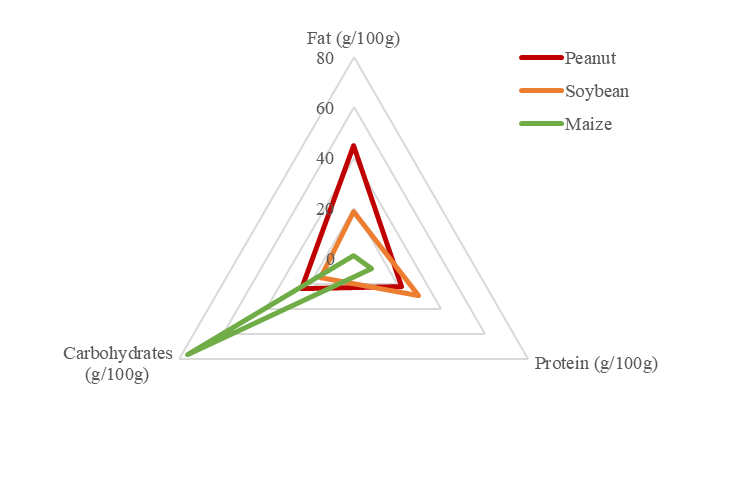


**Figure S5** Intrinsic carbohydrate, protein, and lipid content of peanut, soybean, and maize seeds


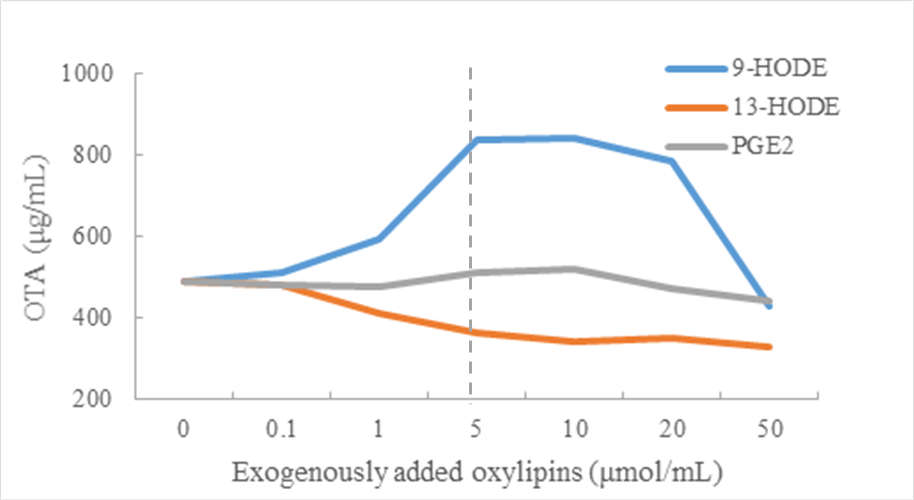


**Figure S6** The application threshold concentration was determined by the effect of oxylipins on OTA. The final application concentration was determined to be 5 μmol/mL, which was on the order of “μg/mL” of *A. ochraceus* auto-secretory oxylipins (9-HODE=1.3 μg/mL and 13-HODE=0.5 μg/mL at the density of 10^3^ spores/mL), and can significantly affect the OTA yield of *A ochraceus*.

**Table S1** Primers used to construct mutant strains

| **Primer** | **Sequence (5’-3’)** | **Function** |
| --- | --- | --- |
| Ao *gprC*-up-F | GACTCGGGCGAGAGAAGATG | To amplify the upstream homologous fragment of Ao *gprC* |
| Ao *gprC* -up-R | GCACTCGTCCGAGGGCAAAGGAATAGAGTAGCAGTTCTCTCCCAATCATCCTGG |  |
| Ao *gprC* -down-F | AACCAAAATAGGCATTGATGTGTTGACCTCCCCTTGAAGTCCGGTGCTCAC | To amplify the downstream homologous fragment of Ao *gprC* |
| Ao *gprC* -down-R | CGAGTATGGAGATGGTGAGGG |  |
| N- Ao *gprC* -F | CATACTCCGACATACCCCTCG | To amplify the Ao *gprC* cassette fusion fragment |
| N- Ao *gprC* -R | CGTCTAAGCTCTCACGGTCAG |  |
| Ao *acyA*-up-F | CTGAAGGAACTCGACGCCAG | To amplify the upstream homologous fragment of Ao *acyA* gene |
| Ao *acyA* -up-R | CTGCTCTTCTCGTCGCAATCG |  |
| Ao *acyA* -down-F | GGGCTCTATGCCACTCATAAGC | To amplify the downstream homologous fragment of Ao *acyA* |
| Ao *acyA* -down-R | CAGTAGAGGGAGTCATGTCGC |  |
| N- Ao *acyA* -F | GTCTGGAGCAGTTGTCGGTC | To amplify the Ao *acyA* cassette fusion fragment |
| N- Ao *acyA* -R | GGCGTGAAGGAATCAGCGAC |  |


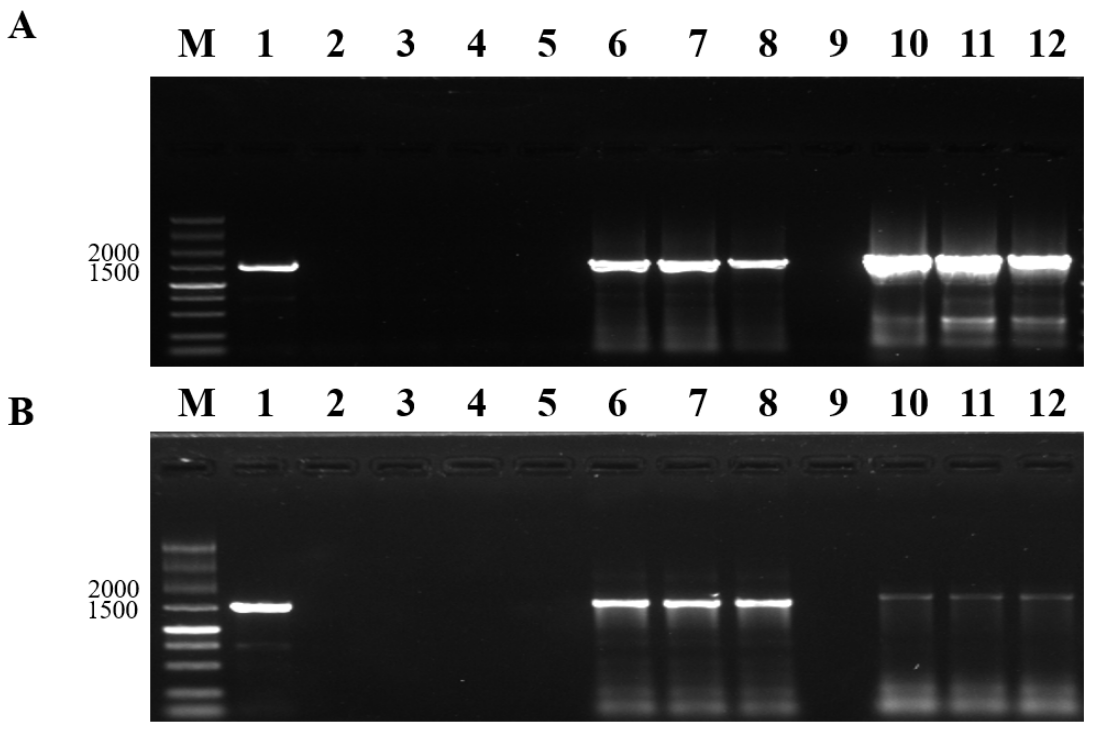


**Figure S7** PCR verified the gene knockout mutants, Δ*gprC* (top) and Δ*acyA* (bottom). The knockout box was introduced into *Aspergillus ochraceus* by PEG-mediated protoplast method, and the knockout mutants was cultured by intracellular homologous recombination. The positive clones were screened and identified: target gene (knockout, KO), upper homologous arm + upstream sequence of hyg (P2) and down homologous arm + downstream sequence of hyg (P3). In wild type strains, KO fragment could be amplified (lane 1), but P2 and P3 fragment could not be amplified (lane 5,9). In mutants, the KO fragment cannot be amplified (lane 2,3,4), but P2 and P3 fragment can be amplified (lane 6,7,8,10,11,12). M:5000marker.
